# Evolutionary design of machine-learning-predicted bulk metallic glasses†

**DOI:** 10.1039/d2dd00078d

**Published:** 2023-01-04

**Authors:** Robert M. Forrest, A. Lindsay Greer

**Affiliations:** a Department of Materials Science and Metallurgy, University of Cambridge UK rmf48@cam.ac.uk

## Abstract

The size of composition space means even coarse grid-based searches for interesting alloys are infeasible unless heavily constrained, which requires prior knowledge and reduces the possibility of making novel discoveries. Genetic algorithms provide a practical alternative to brute-force searching, by rapidly homing in on fruitful regions and discarding others. Here, we apply the genetic operators of *competition*, *recombination*, and *mutation* to a population of trial alloy compositions, with the goal of evolving towards candidates with excellent glass-forming ability, as predicted by an ensemble neural-network model. Optimization focuses on the maximum casting diameter of a fully glassy rod, *D*_max_, the width of the supercooled region, Δ*T*_x_, and the price-per-kilogramme, to identify commercially viable novel glass-formers. The genetic algorithm is also applied with specific constraints, to identify novel aluminium-based and copper–zirconium-based glass-forming alloys, and to optimize existing zirconium-based alloys.

## Abstract 2

We use a genetic algorithm driven by a neural-network to efficiently search for glass forming alloy candidates.

## Graphical abstract

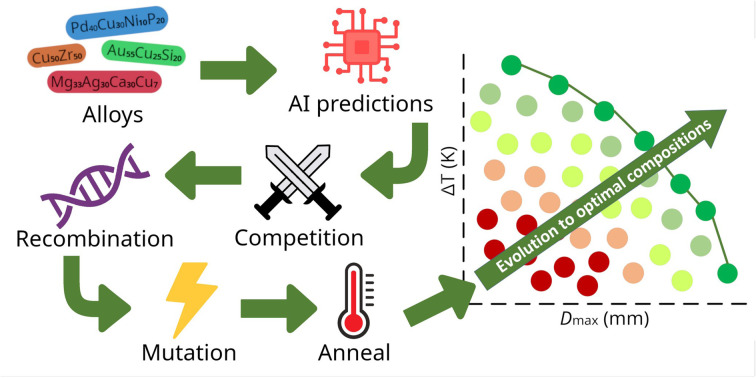

## 1 Introduction

*Metallic glasses* (MGs) are a subset of amorphous materials. They lack the periodic long-range ordering found in crystalline materials,^1^ and are formed when a liquid metal alloy is cooled sufficiently quickly to deny time for significant growth of crystalline nuclei. Instead, the liquid structure is locked into the solid state.^2^ Distinctions can be made within MGs, defining *bulk* metallic glasses (BMGs) as those that can be obtained in samples with a minimum thickness of 1–10 millimetres, rather than thin glassy ribbons (GRs) or powders.^3,4^

The *glass-forming ability* (GFA) of an alloy composition refers to its ability to resist crystallization during cooling. Two measurable quantities that are directly related to the GFA are *D*_max_, the maximum castable diameter of a fully glassy rod,^5^ and the critical cooling rate, the minimum rate at which a liquid must be quenched to avoid crystallization.^6^ Compositions with higher GFA can be quenched at lower rates and cast to larger diameters, leading more readily to use in commercial applications.

The lack of an atomic lattice and accompanying defects,^2^ the significant effects of which dominate the properties of crystalline materials,^4^ results in MGs having interesting properties, including excellent soft-magnetism, corrosion resistance, hardness, yield strength, and resilience.^7^ MGs typically have thermal expansion properties similar to crystalline materials, lower values of thermal conductivity, and undesirably low ductility and castable diameter.^7^

There are many potential applications for MGs, ranging from sporting goods^8^ and smartphone components,^9^ to precision gears^10^ and fuel-cell separators,^11^ among others.^12^ High hardness observed in many BMGs enables application in scratch-resistant fashion items, such as jewellery and watches.^13^ In comparison with other biomaterials, the mechanical properties of zirconium-based BMGs are better matched to those of bone,^14^ and are potentially biocompatible,^15^ making them promising implant candidates. The application of metallic glasses to functional and structural problems is limited by the difficulty in discovering alloys with both properties meeting the requirements of the design, and high GFA allowing casting to sufficient dimensions.

The wide variety of applications of MGs are merely those that have been imagined so far, for the MG-forming compositions that have been discovered. It is certainly the case that there are vast regions of composition-space not yet probed, containing glass-forming compositions suitable for applications so far unconsidered. Data-driven materials discovery is a promising tool to both explore and exploit the field of metallic glasses.

**Fig. 1:**
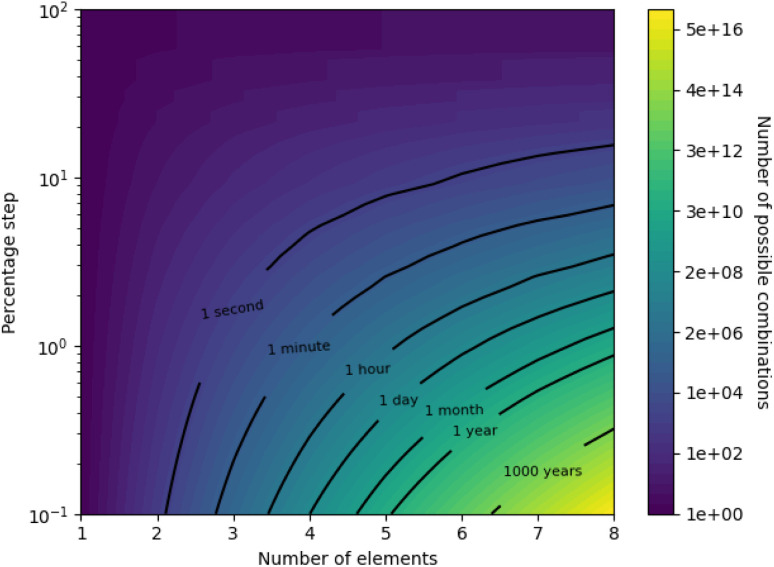
Contour map showing the size of composition-space under the constraints of the number of elements in an alloy, and the allowed percentage step. Note the logarithmic scale of the contour colouring. Particular isochrones are labelled with the approximate time required to compute predictions for that many alloy compositions, assuming a rate of 2000 compositions-per-second.

Due to the size of composition space, illustrated in Table 1 merely *via* equiatomic alloys, a simple grid-based search (wherein possible alloy compositions are stepped through one by one) is infeasible unless tightly constrained. This is further illustrated by Fig. 1, which presents a contour map of the number of possible alloy compositions given the number of elements in an alloy and the allowed percentage increment, on a logarithmic scale. It is apparent from Fig. 1 that a high-definition study of all possibilities in an 8-element alloy is infeasible computationally, let alone experimentally, as there are on the order of a hundred quadrillion valid combinations of 8 elements with a percentage step of 0.1%. Furthermore, specific isochrones on the map are labelled with the approximate amount of time it would take to compute predictions for that many alloy combinations, assuming a rate of 2000 combinations per second (measured from our model on typical desktop hardware) and serial calculation. Taking a reasonable limit of feasibility to be a month of constant computation, this places rough restrictions on current abilities with brute-force searching to a range between high-definition studies of 4-element alloys and low-definition studies of 8-element alloys. While the computation time may be reduced by more powerful computer hardware, or parallelized searching, the logarithmic nature of Fig. 1 requires exponential acceleration to meaningfully push the boundary of feasibility. Probing the high-definition space of an 8-element alloy would currently take around 3 million years, making this feasible would require a multi-million factor acceleration – something that the approaching failure of Moore's law^16^ suggests to be unlikely.

**Table 1:** Number of possible equiatomic alloys as a function of the number of elements in an alloy, calculated from ^*n*^*C*_*r*_ where *n* = 118 and *r* = the number of constituent elements, giving a lower-bound on the size of the composition-space and illustrating the rapid expansion of the search-space when considering alloys with more constituent elements

Number of constituent elements	Possible equiatomic alloys
1	118
2	6903
3	266 916
4	7 673 835

It is essential that alternative approaches to materials discovery are used to avoid this problem. While historically trial-and-error has generated most innovations, this strategy cannot continue in the long term, particularly as material complexity increases and serendipity must contend with many degrees of freedom.^17^*Genetic algorithms* (GAs) use techniques analogous to processes observed in evolutionary biology to find good solutions to a given problem, with potentially far greater efficiency than a grid search, and are discussed further in Section 2.

In our previous work^18^ we trained a novel ensemble neural-network (NN) model capable of simultaneously predicting several properties of any given alloy composition, including the probability of glass-formation and the maximum castable diameter of a fully glassy rod, *D*_max_. To provide background, this model is briefly discussed in Section 1.1.

In the present work, a genetic algorithm is used to iteratively search for compositions that optimize desired properties as predicted by the neural-network model, both with constraints, optimizing existing alloys, and without constraints, freely searching for novel alloys. GAs have been established for some time as a powerful tool for materials science,^19,20^ in particular being frequently used to optimize the properties of existing materials,^21,22^ the parameters of processing techniques,^23,24^ and to design entirely novel materials.^25,26^ GAs have also been applied in the search for novel glass-forming alloy compositions; Bansal *et al.*^27^ applied a NN and GA to identify glassy structures in Cu–Zr alloys with good resistance to shear deformation, Sun *et al.*^28^ used a GA to search for energetically favoured packing motifs in glassy Cu–Zr and Al–Sm alloys, and Tripathi *et al.* applied genetic programming to identify glass-forming ability criteria.^29,30^

Three main objectives are considered in the present work's search for new glass-forming alloy compositions: maximization of *D*_max_ and Δ*T*_x_ (=*T*_x_ − *T*_g_), and minimization of the cost-per-kilogramme. *D*_max_ is a direct measurement of the GFA of an alloy, while Δ*T*_x_ measures the width of the supercooled region upon heating, and is related to the stability of the glass.^31^ Both these quantities may be maximized to return higher GFA, and they are considered simultaneously here to provide two different metrics of GFA.

While a higher inherent GFA would improve the possibility of a glass-forming alloy being reliably cast to sufficient dimensions for use in real-world applications, high-GFA compositions often include expensive elements among their constituents,^32^ thus raising commercial barriers. For this reason, the price-per-kilogramme is investigated as a minimization objective in this work. The optimization of these three objectives is intended to identify commercially viable BMG-forming alloys, capable of feasibly being incorporated into product designs.

### 1.1 Model for glass-forming ability

In our recent work^18^ we trained a machine-learning model of glass-forming ability. The open-source machine-learning framework *Tensorflow*^33^ was used to construct and train neural-network models to predict the following properties of alloys:

• The liquidus temperature *T*_l_,

• The temperature of the onset of crystallization *T*_x_,

• The glass-transition temperature *T*_g_,

• Classification of GFA as crystalline, GR, or BMG,

• The maximum castable diameter of a fully glassy rod *D*_max_.

The networks consisted of a number of densely linked hidden layers of neurons, and contained multiple output neurons, allowing a single model to be trained to predict all of the desired properties. The models were trained on a dataset consisting of data on pure elements, and experimental measurements from investigations of glass-forming alloys, compiled from previous publications attempting to model GFA.^34,35^ Of the compositions in the training dataset, 1700 (25.6%) were crystalline, 3763 (56.7%) were GRs, and 1175 (17.7%) were BMGs. All GRs were approximated to have a *D*_max_ of 0.15 mm, following literature precedent.^35^

Our previous work concerning the neural-network model for GFA focused on extracting useful theoretical insights into glass formation, by studying the reliance of the model itself on the supplied data inputs. The present work takes an alternative approach, instead probing the outputs of the model by means of a GA.

### 1.2 Model uncertainty

Typical neural-network models do not report on the uncertainty they have in their predictions. Access to uncertainty information is highly desirable, as it enables evaluation of the quality of individual predictions, rather than having to rely on dataset-wide performance metrics, such as the mean-absolute-error of the model, which are useful only for in-domain predictions.

Gal and Ghahramani^36^ proposed the use of dropout layers,^37^ which randomly ‘drop’ neuron connections with the intention of promoting generalisation rather than overfitting, as a route to quantifying uncertainty in standard neural networks. Generally, dropout layers are active only during training, and are disabled when a model is used for inference. If the dropout layers are not disabled, and neuron connections are randomly removed during inference, different predictions may be made by the model for the same input – the variation across many repeated predictions is interpreted as the uncertainty of the model for that specific input. Since our ensemble model contains dropout layers, we are able to quantify the model's uncertainty in all predictions made *via* the method of Gal and Ghahramani. Each prediction made here is repeated 100 times with dropout layers active, taking the mean as the final value, and the 95% confidence interval as the uncertainty.

## 2 Evolutionary algorithms

Evolutionary computing applies the concept of evolution by natural selection to the process of finding optimal solutions to a problem.

**Fig. 2:**
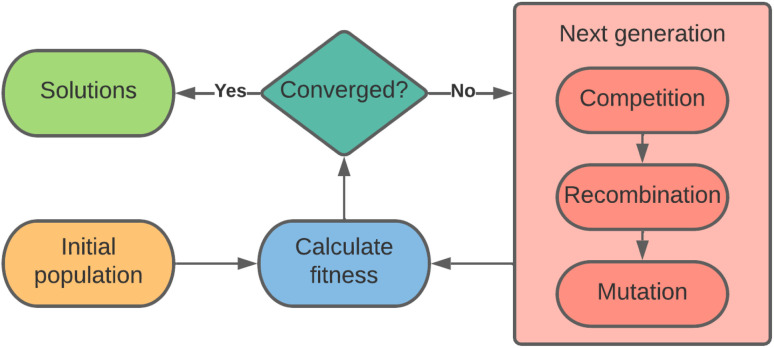
Illustration of the flow of a genetic algorithm. A population of candidate solutions is acted upon by *competition*, *recombination*, and *mutation* operators to produce a new generation of solutions, which are assessed on their fitness in relation to the objectives.

Fig. 2 illustrates the general flow of a genetic algorithm, wherein a population of candidate solutions is assessed on their *fitness*, and acted upon by *competition*, *recombination*, and *mutation* operators, each described in the following sections, until there is convergence on a solution.

### 2.1 Fitness

The biological interpretation of *fitness* refers to the ability of an organism to reproduce and propagate genetic material to the next generation. In evolutionary computing the same is true, as fitness is determined by the degree that a candidate solution fulfils the objectives of the scenario, and is the metric by which the selection process for the next generation is guided.

There are various methods by which fitness can be defined to enable calculation. For example, if an investigation is searching for glass-forming alloy compositions with large *D*_max_ values, fitness could be defined simply as the value of *D*_max_. The situation becomes more complex when there are multiple objectives, as the ability of a candidate must be assessed on each criterion to provide an overall judgement that is comparable to judgements made about its peers. The criteria a candidate is being judged on may have wildly different scales, ranges, and interpretations, such that their raw numerical values cannot be simply compared or combined to give a total score.

#### 2.1.1 Pareto frontiers

Multi-objective optimizations give rise to a *Pareto frontier*,^38^ defined by the points at which no objective can be improved upon without detrimental impact on the others, giving the set of solutions described as *Pareto optimal*.^39^ Candidates may be judged under multiple criteria using the method of *Pareto ranking*, also called non-dominated sorting,^40^ whereby the population is ordered by the Pareto frontiers in which they appear. For example, the first frontier, or rank, contains those candidates that Pareto-dominate all other candidates. The second rank contains candidates that Pareto-dominate all candidates outside the first rank, and so forth, with the *N*^th^ rank containing candidates dominating all others not contained within the preceding (*N* − 1) ranks.

Pareto ranking allows the comparison of the fitness of any two candidates in different frontiers. To allow ranking of candidates found in the same frontier, and in the interest of improving diversity within the population, algorithms may compare candidates by their average similarity to the entire population of the frontier, favouring those that are more different. One such measure of diversity is the *crowding distance*, calculated as the distance along a frontier to the neighbours of a candidate, averaged over each direction. Candidates with a shorter crowding distance are in a region of higher population density, and are less preferable than candidates with a longer crowding distance. This measure is used by the popular NGSA-II evolutionary algorithm,^41^ which itself is used as the template for the algorithm implemented in this work. It is noted that the crowding distance is not useful for measuring the objective fitness of a candidate; rather it is a metric that prevents algorithms entering local minima with tunnel vision.

### 2.2 Alloy candidates

In this work, each alloy candidate is defined as an associative list, that being a data-structure defined by key-value pairs. Each element in an alloy composition is a ‘key’ linked to the percentage of that element in the composition, the ‘value’. Throughout, the percentage of an element in an alloy composition refers to the atomic percentage, rather than the weight percentage. Multiple instances of this alloy data-structure with different elements and percentages form the overall population acted on by the GA; examples of trial compositions may include the following:

{(Cu, 75%), (Zr, 15%), (Al, 9.5%), (Ti, 0.5%)}

{(Be, 50%), (U, 50%)}

{(Li, 99.9%), (Fe, 0.1%)}

{(Pd, 100%)}

The initial population of alloy candidates is generated entirely randomly, with no requirement for realism, such that alloys that could never be created may be suggested. It is the subsequent action of the GA that transforms the random population into feasible BMG-formers.

### 2.3 Competition

The *competition operator* acts by holding tournaments among the population.^42^ Random pairs of compositions are drawn from the population and compared; the fitter candidate wins and proceeds to the next stage, no longer needing to compete, while the losing composition is returned to the gene pool to compete in further tournaments. Tournaments continue until the desired percentage of the existing population has been selected. In this work, 50% of the population is selected, and the remaining compositions, which did not win any tournaments, are discarded as victims of natural selection.

Tournaments are held, rather than simply selecting the elite top 50% of the population, in order to decrease the likelihood of the algorithm becoming trapped in local minima.

### 2.4 Recombination

The *recombination operator* acts on tournament-winning compositions. Two ‘parent’ compositions, *A* and *B*, are randomly selected from the successful gene pool and are mixed using the technique of intermediate recombination,^43^ whereby two ‘child’ compositions, *A*′ and *B*′, are determined as,1
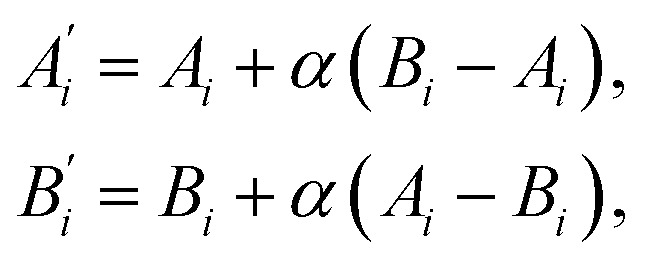
where *A*_*i*_ is the atomic percentage of element *i* in composition *A*, and *α* is a random number between 0 and 1. The newly generated child compositions replace their parents in the gene pool, conserving the overall population size. This method of recombination allows for ‘clones’ of parents to enter the next generation when the value of *α* is small.

The intermediate recombination approach is used here, rather than a crossover-based approach, because the genes of a composition have real-valued rather than integer representations, being the value of the atomic percentage of each element.

The *recombination rate*, the probability that parent compositions undergo recombination rather than simply being copied into the next generation, is set to the commonly used value of 90%.^44^

### 2.5 Mutation

The *mutation operator* acts on the ‘child’ compositions produced during the recombination stage, each individually having a chance of mutation. This probability, referred to as the *mutation rate*, is required to be low to ensure the algorithm does not degrade to a random search,^45^ and here is set to 5% per child per iteration.^44^

During mutation, the genetic code of an alloy composition undergoes one of several possible alterations:

• *Add*: a new element is added to the composition with a random percentage:
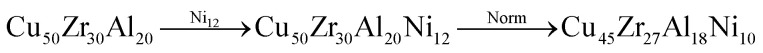


• *Remove*: an element is removed from the composition:
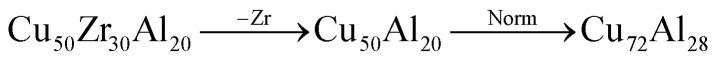


• *Swap*: the percentages of two elements in the composition are swapped:
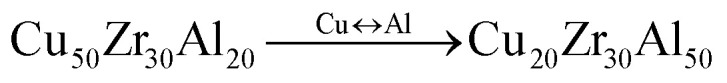


• *Adjust*: the percentage of an element in the composition is changed by a random amount:
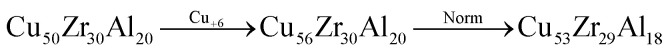


After all kinds of mutation, the percentages in a trial alloy composition are rescaled to ensure they sum to 100%. Any elements with a percentage below a threshold of 0.01% are removed from the composition. The culling of trace elements from compositions is performed to ensure composition percentages remain within the level of fine control typically possible during production – alloys with ≤0.01% of impurities are referred to as “super pure”.^46^ Limiting the minimum atomic percentage in the candidate alloys decreases the size of the search space, as illustrated by Fig. 1, meaning the algorithm does not waste effort investigating alloys containing element traces that could not be reliably replicated experimentally.

### 2.6 Constraints

Brute-force approaches to materials discovery, such as a grid search through elemental percentages, are not feasible beyond highly constrained problems due to the practically boundless size of composition-space. The GA approach enables ‘smart’ exploration of the space, reducing the time to solution by avoiding less fruitful regions and homing in on areas of interest.

However, due to the stochastic nature of GAs and the lack of a single well-defined solution for the ‘best glass-former’ led to by smooth gradients, the alloy compositions selected are highly sensitive to the initial guesses. Given that these initial guesses are also random, the algorithm will generally return novel suggestions each time it runs, as it explores different areas of the composition-space and converges upon different local minima. This effect becomes more pronounced as the size of the accessible composition-space, *i.e.* the number of elements allowed in a trial composition, increases. While this can be mitigated by techniques such as *simulated annealing*, as discussed in Section 2.7, algorithmic investigation can be guided to areas of existing human interest *via* the application of constraints to the trial compositions. For example, certain elements may be required in every composition, with upper or lower bounds on their percentages. Such constraints also allow the GA to be employed in the optimization of existing alloy compositions, by limiting elemental percentages to be close to the nominal values of a particular alloy.

Restrictions on alloy compositions are applied here through an additional genetic operator, the *constraints operator*, which acts on every composition in the population. Given a set of rules, the constraints operator modifies any composition which violates a rule, until all rules are satisfied. For instance, if a rule specifies that the element copper must be present in every candidate, with atomic percentage between 40% and 60%, the constraints operator would act in the following way for these example alloy compositions:
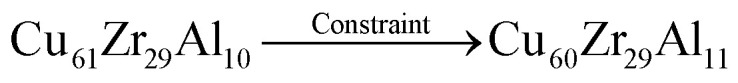

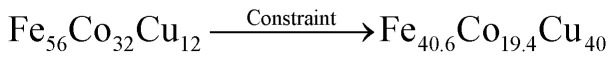

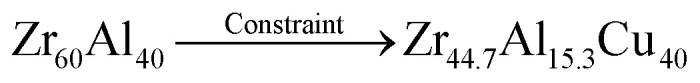


An alternative approach to applying constraints, not used here, is to penalise the fitness score of illegal candidates, leading to evolution towards candidates that both have the desired properties and exist within the constrained search-space.^47^ The constraints operator has the benefit, in comparison to penalisation, of effectively reducing the size of the search-space, as the illegal candidates are inaccessible.^48^ Conversely, the constraints operator is somewhat more complex to implement than a simple penalisation of candidate fitness.

### 2.7 Simulated annealing

Simulated annealing (SA) is, like the genetic algorithm, a search technique that draws inspiration from the real world – in this case, annealing is in reference to the metallurgical process of subjecting a material to controlled heating and cooling, providing thermal energy to promote the removal of crystal defects by relaxation out of local energy-minima.^49^

Given an existing candidate, A, and a new candidate B, with fitnesses *f*_A_ and *f*_B_, SA selects a candidate using the following scheme:^50^2
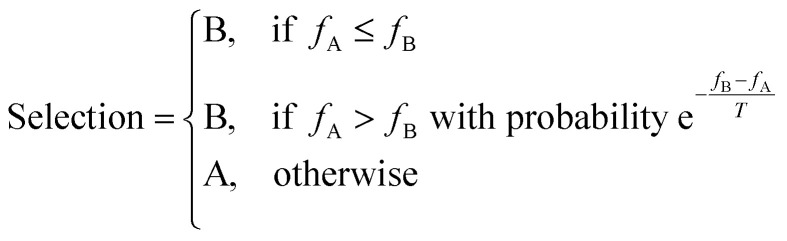
where *T* is the ‘temperature’, which scales the probability of selecting the new candidate even when it exhibits poorer performance than the original. High temperatures provide the search process with ‘energy’ to escape from local fitness-minima in which other search algorithms, such as GAs, may become trapped. This ‘hill climbing’ ability of SA results in a better chance of obtaining globally optimal solutions, but with the trade-off of its heavily stochastic nature meaning convergence can take longer to achieve.^50^

The annealing temperature is reduced over the course of the search by means of a *cooling schedule*, of which there are a large variety. Cooling quickly may prematurely cull the algorithm's freedom to widely explore the search space, while cooling slowly may inefficiently prolong the computation time required to obtain good solutions.^51^

In this work, we incorporate SA as an additional stage of the GA, resulting in a hybrid search scheme able to benefit from the exploratory capabilities of SA.^52^ Here, alloy candidates of the current generation, created by the action of the competition, recombination, and mutation operators, are compared during annealing against randomly generated alloys. We apply the commonly used geometric cooling schedule, which defines the annealing temperature as,^51^

3 *T*_*i*_ = *T*_0_*α*^*i*^,

where *T*_0_ is the initial temperature, here 500, *α* is the cooling rate, here 0.9, and *i* is the current generation.

### 2.8 Convergence

Since we do not have *a priori* knowledge of the globally optimal solutions to the problem of identifying good glass-forming alloys, we cannot know with complete certainty whether the GA has identified these optimal solutions. The solutions returned may merely be optimal locally to the current state of the algorithm, rather than globally optimal throughout the entire search space. The ability of a GA to obtain globally optimal solutions depends on the balance of *exploration*, which involves entering new areas of the search space not previously probed (global searching), and *exploitation*, the use of currently known good solutions to attempt to obtain similar but improved solutions (local searching).^53^ Of the previously mentioned genetic operators, exploration is performed by the mutation operator and simulated annealing, and exploitation by the recombination operator.^45^

*Convergence* in scientific computing generally refers to the state in which no further improvements upon the current best solution can be found. The likelihood that the current best solution is globally optimal is related to the quality of the searching algorithm, and the *convergence criterion* used to decide whether searching continues or terminates. Here, we define convergence as 20 consecutive generations with less than 1% improvement on each of the target objectives.

### 2.9 Novelty of alloy candidates

It is well established that machine-learning models are less effective when used to make predictions outside the domain of their training data – that is, to *extrapolate* rather than *interpolate*.^54–56^ As such, it is useful to quantify the similarity between a new alloy candidate generated by the GA, and the original dataset of alloys used to train the GFA model.^18^

The *local outlier factor* (LOF) of Breunig *et al.*^57^ measures the relative isolation of an object in its local neighbourhood, outlying objects being those existing in areas of the space with substantially lower density,4
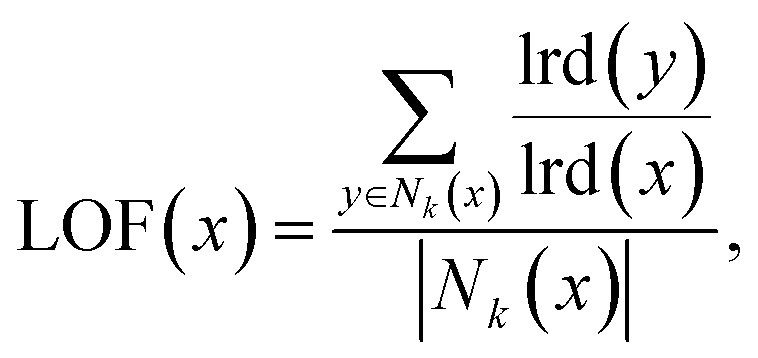
where *x* is the object being measured for novelty, *N*_*k*_ is the set of *k* nearest neighbours to *x*, and lrd(*x*) is the *local reachability density*, defined as,5
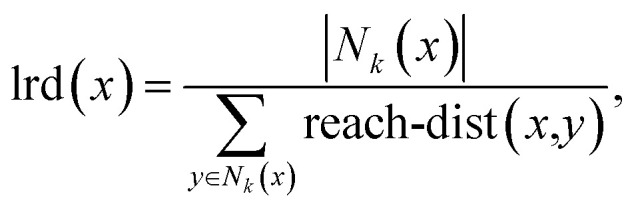
where reach-dist(*x*,*y*) is the *reachability distance* between *x* and a neighbour *y*,

6 Reach-dist(*x*,*y*) = max[*k*-distance(*y*),*d*(*x*,*y*)],

where *d*(*x*,*y*) is a distance metric between *x* and a neighbour *y*, and *k*-distance(*y*) is the distance to the *k*^th^ nearest neighbour. Large values of LOF(*x*), >1, indicate that *x* is an novel outlier, while small values, <1, indicate that *x* exists within an existing cluster of data.

While the LOF was originally intended for outlier detection within datasets, it can equivalently be considered as a measure of the ‘novelty’ of a new data-point with respect to an existing dataset.^58,59^ To ensure that the machine-learning model for GFA is used meaningfully, for interpolation rather than extrapolation, alloy candidates identified by the LOF to be outliers are discarded in this work. In other investigations, where on-the-fly data gathering and model re-training is possible, outlying candidates provide a valuable opportunity to expand the experience of the model and reinforce its understanding.^60–62^ We welcome the experimental validation of all the BMG-forming alloy candidates identified here by the GA, data on which, positive or negative, would allow updating of the training dataset and re-training of the model.

As the number of dimensions increases, the *relative contrast* of a distance measure, being the difference between the shortest and longest lengths, tends towards zero.^63^ Thus, the ability of a distance measure to distinguish objects in high-dimensional spaces also tends towards zero – this phenomenon is known as the ‘curse of dimensionality’.^64^ The feature-space of the alloy candidates in this work is 39-dimensional,^18^ and so to mitigate the curse, we apply principal-component analysis (PCA), enabling embedding of samples in a three-dimensional space where distance measures remain meaningful,^65^ before calculation of the LOF.

## 3 Evolved alloy compositions

### 3.1 Maximization of *D*_max_

**Fig. 3:**
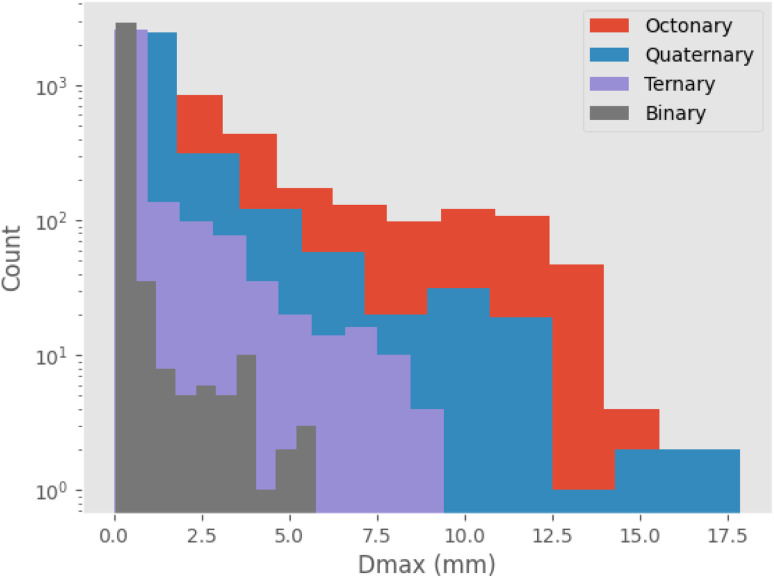
Logarithmic histogram of predicted *D*_max_ values across the entire population of alloy compositions considered by the genetic algorithm when considering binary (two-element), quaternary (four-element), quinary (five-element), and octonary (eight-element) alloys. The rate of increase of maximum *D*_max_ is observed to slow as more elements are included in alloys.

**Fig. 4:**
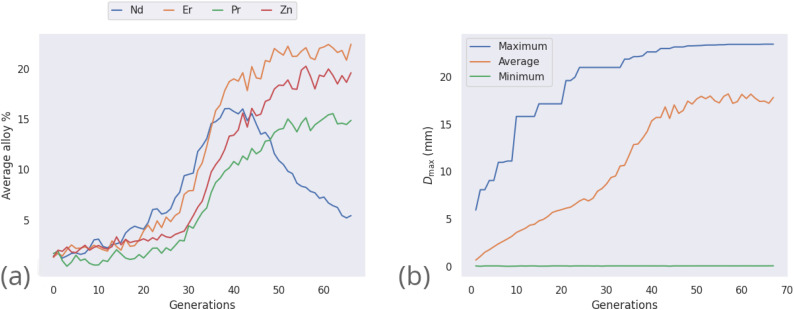
Progress of the genetic algorithm while maximizing *D*_max_, showing (a) the average percentages of elements in the population of alloy candidates for those which are observed above 5%, and (b) the maximum, average, and minimum values of predicted *D*_max_ across the population of alloy candidates.

Initially, maximization of *D*_max_ alone is considered, to gain insight into high-*D*_max_ compositions without needing to consider the simultaneous impacts of Δ*T*_x_ and price optimization. Table 2 lists several binary, ternary, and quaternary compositions found by the GA to have large predicted *D*_max_ values. As is further illustrated by Fig. 3, larger *D*_max_ values are exceedingly rare, but as would be expected from previous insights such as Inoue's rules^66^ and the confusion principle,^67^ a wider range of *D*_max_ is accessible by increasing the number of constituent elements in the trial alloy compositions. The increase in observation counts at the high-value tail of the distributions, seen particularly with the octonary alloys, is due to the action of the GA specifically promoting large values and eliminating others. While increasing the number of constituent elements in the glass-forming alloys does initially have a large impact on the range of GFA accessible, diminishing returns are observed; a smaller increase in maximum *D*_max_ is gained when moving from quinary to octonary alloys than from binary to quaternary. The action of the GA is illustrated by Fig. 4, showing the increase in predicted *D*_max_ over the evolutionary process and the associated changes in atomic percentages.

**Table 2:** Sample of alloy compositions identified by the genetic algorithm to have large predicted *D*_max_ values, selected from binary, ternary, and quaternary results. Confidence intervals calculated over 100 repeated model predictions with dropout layers active. Negative local outlier factors indicate that a candidate exists within a cluster of the original training data

Composition	Predicted *D*_max_ (mm)	Local outlier factor
Y_41_Er_36_Be_13_Rh_10_	17.2 ± 3.4	−0.45
Zn_39_Y_27_Nd_17_Yb_16_	16.8 ± 3.1	−0.47
Er_42_Mg_25_Be_18_Cu_15_	11.8 ± 2.6	−0.48
Co_32_Y_30_Ho_21_Ba_17_	11.2 ± 1.6	−0.52
Y_38_Ag_36_Dy_26_	9.1 ± 1.6	−0.51
Ca_40_Mg_37_Cu_23_	5.6 ± 1.0	−0.48
Ho_41_Cu_31_Zr_15_Sc_13_	5.5 ± 0.4	−0.53
Dy_44_Mn_33_Li_17_Be_6_	5.4 ± 1.0	−0.46
La_39_Tm_36_Rh_25_	3.8 ± 1.0	−0.47
Zn_63_Tm_37_	2.9 ± 1.2	−0.31
Yb_52_Zn_48_	2.2 ± 0.9	−0.48
Al_68_Yb_32_	0.9 ± 0.3	−0.47

**Fig. 5:**
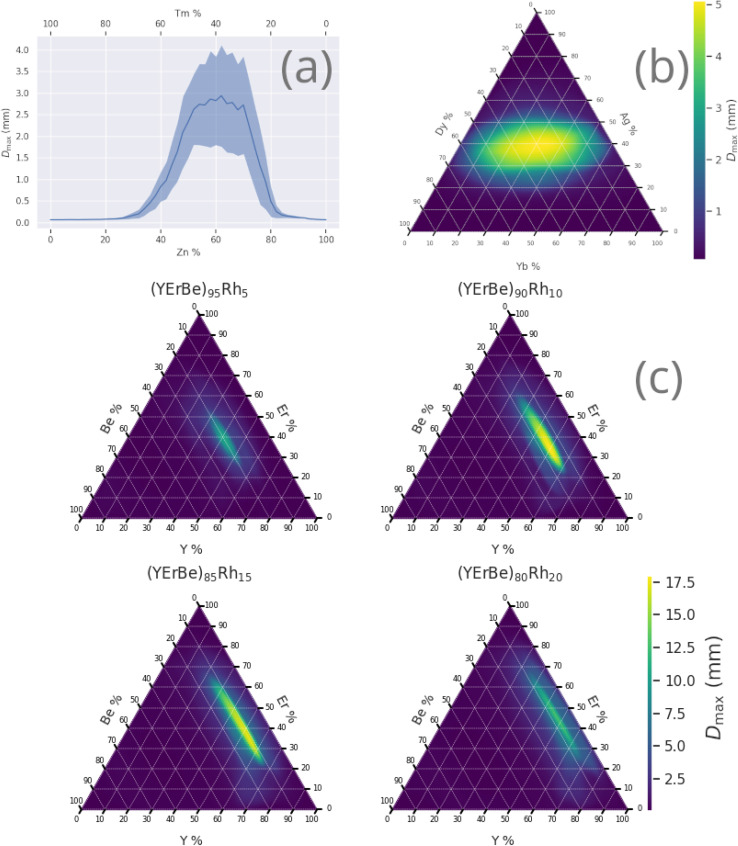
Prediction of *D*_max_ across composition-spaces containing specific compositions identified by the genetic algorithm to have high *D*_max_, (a) Zn–Tm, (b) Yb–Ag–Dy, (c) Y–Er–Be–Rh. Shaded region of binary diagram denotes the 95% confidence interval. Ternary diagrams may be read by following the grid-lines in the direction of the axis ticks; for example, in (c) the horizontal lines are of constant erbium content.

Fig. 5 shows the variation of *D*_max_ within various alloy compositions, illustrating the drastic changes in GFA that can occur across the composition-space of a single alloy, often over relatively small percentage ranges.

### 3.2 Simultaneous maximization of *D*_max_ and Δ*T*_x_, and minimization of price

Many high-GFA alloy compositions include expensive elements; indeed the average cost-per-kilogramme of glass-forming alloys in the dataset used in our previous work^18^ was $2310, while this was only $660 for crystal-formers. The price of an alloy, *P*, is calculated from the prices of the constituent elements *via* the linear mixture,

7 *P* = ∑*w*_*i*_*p*_*i*_,

where *w*_*i*_ is the percentage by mass of element *i* in the composition, and *p*_*i*_ is the price-per-kilogramme of that element. The percentage by mass is calculated from the atomic percentage, atomic mass, and the total atomic mass,8
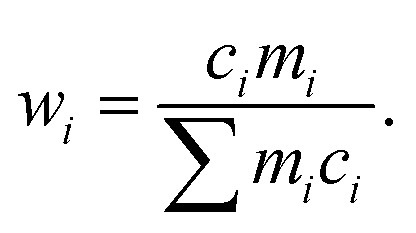


This approach is the same as that used in our previous work and throughout the literature to calculate approximate properties of alloys based on elemental information.

Pricing data used here are not intended to be highly accurate or recent; indeed due to market fluctuations it would be impossible to avoid immediate obsolescence. Instead, these data merely act as a reasonable proxy for the commercial barriers faced when attempting to manufacture an alloy, such as supply-chain maturity and material availability. The pricing data were acquired from multiple sources reporting on several different years of market activity.^68–71^ Pricing data for all elements are given in ESI S1.†Table 3 presents a sample of compositions identified by the GA to simultaneously have large predicted *D*_max_ and Δ*T*_x_ values and low price-per-kilogramme.

**Table 3:** Sample of compositions identified by the genetic algorithm to have simultaneously large predicted *D*_max_ and Δ*T*_x_ values and low price-per-kilogramme

Composition	*D*_max_ (mm)	Δ*T*_x_ (K)	Price ($ per kg)
Fe_44_B_22_Ce_18_V_16_	5.14 ± 1.28	67.3 ± 7.72	54.3
B_30_Mn_28_Ba_27_Co_15_	6.44 ± 1.81	40.1 ± 4.71	5.26
Er_53_Mg_30_Cu_17_	4.88 ± 0.54	55.2 ± 5.15	22.7
Y_38_Ti_37_B_25_	4.67 ± 1.52	42.3 ± 3.71	23.3
Gd_49_Cu_34_Mg_17_	4.59 ± 0.78	63.1 ± 3.10	22.8
Er_47_Ti_37_B_16_	4.76 ± 0.58	46.7 ± 1.74	23.3
Yb_50_Zn_50_	2.01 ± 0.89	35.5 ± 2.11	13.1
Er_52_Zn_48_	1.24 ± 0.73	41.6 ± 6.70	20.1
Mg_66_Mn_34_	1.47 ± 0.76	37.6 ± 4.49	2.05

The price-per-kilogramme of elements varies over several orders of magnitude, but given the previously mentioned average cost-per-kilogramme of glass-forming alloys of $2310, even the most expensive compositions listed in Table 3, are relatively inexpensive. Some compositions identified by the GA when optimizing multiple objectives also appear in the previous results focusing solely on *D*_max_. For example, the Yb–Zn composition shown in Table 2 is identified again here, albeit with slightly different elemental percentages. This is to be expected since the single-objective problem is a subset of the multi-objective problem – candidates that perform extremely well on one objective, but poorly on the remainder, can nonetheless Pareto-dominate other candidates by nature of there being no other improvements among the population with respect to that objective.

### 3.3 Prediction of specific novel glass-forming alloys

#### 3.3.1 Aluminium-based glass-forming alloys

Aluminium-based BMGs are of interest due to the potential for high strength and corrosion resistance^72^ at low cost, but their creation is hindered by the need for high cooling rates.^73^ Here, we probe the Al-based composition-space to predict novel BMG candidates. The constraints enforced on the trial compositions considered by the GA are only that aluminium must be the element with the highest atomic percentage.

The intrinsic contribution to an alloy property *A* of an element *i* is determined from the composition-weighted average of the property across compositions containing that element from the large set of compositions considered by the GA,9
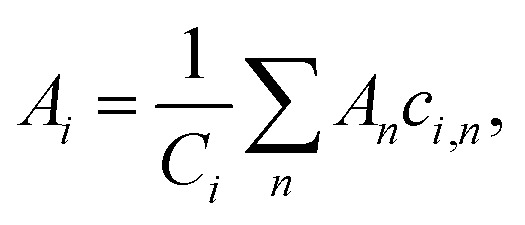
where *c*_*i*,*n*_ is the atomic percentage of element *i* in composition *n*, and *C*_*i*_ is the total sum of atomic percentages of element *i* in the set of compositions. This allows determination of associations between the presence of particular elements and the values of alloy properties, with contributions from elements appearing either in large quantities in a few high-performing alloys, in lower amounts at higher frequencies, or at any point between these extremes. To avoid any bias caused by the initial population supplied to the GA, statistics are calculated across multiple independent evolutions.

**Fig. 6:**
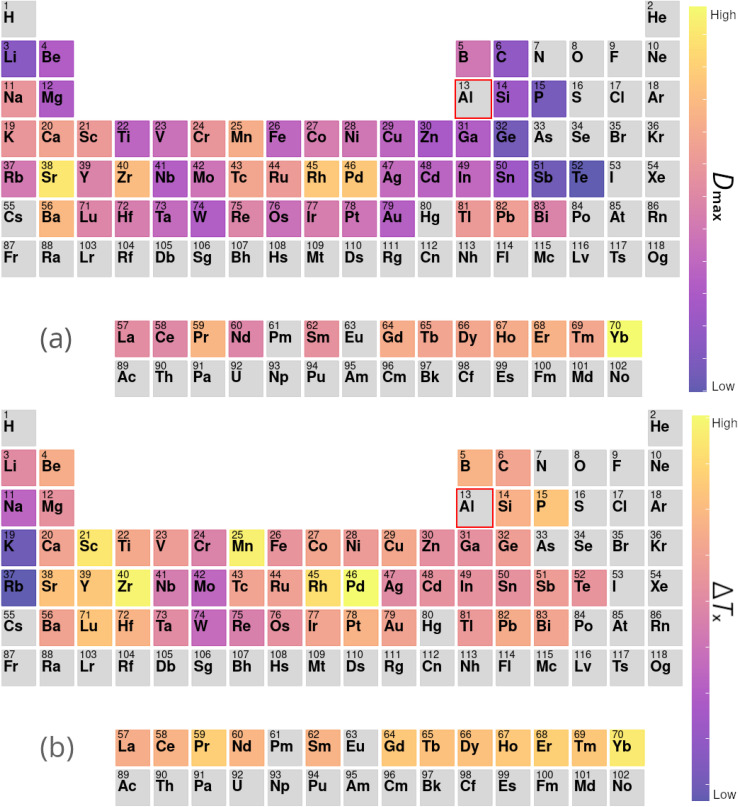
Contributions to (a) *D*_max_ and (b) Δ*T*_x_ of elements added to aluminium-based alloys, calculated from the composition-weighted average, defined in eqn (9), for each element across the alloy populations generated by multiple independent runs of the genetic algorithm.

Table 4 presents a sample of aluminium-based BMG candidates identified by the GA, while Fig. 6 demonstrates the more general impact on glass-forming ability of including other elements in aluminium-based alloys as calculated by eqn (9). It is observed that, generally, the algorithm attempts to lower the percentage of aluminium in candidate alloys to increase the GFA, and finds particular success alloying Al with Pd, Mn, Zr, Sc, Sr, and the lanthanides. However, elements such as Pd and Sc are extremely expensive, and as such are avoided by the GA despite their contributions to GFA. A sample of high-GFA Al-based alloy candidates identified by the GA, but penalised because of their expense, is given in Table 5.

**Table 4:** Sample of Al-based compositions identified by the genetic algorithm to simultaneously have large predicted *D*_max_ and Δ*T*_x_ values and low price-per-kilogramme

Composition	*D*_max_ (mm)	Δ*T*_x_ (K)	Price ($ per kg)
Al_32_Gd_32_Ho_23_Be_13_	6.69 ± 3.1	60.4 ± 5.0	47.3
Al_41_Yb_37_Er_12_Te_10_	3.39 ± 0.61	60.1 ± 2.75	22.6
Al_51_Yb_32_Cr_17_	4.04 ± 0.43	59.4 ± 4.89	13.6
Al_62_Yb_30_Ca_8_	2.13 ± 1.6	48.6 ± 3.56	12.9
Al_54_Yb_40_Ba_6_	2.96 ± 1.4	47.9 ± 2.68	13.1
Al_62_Yb_38_	0.67 ± 0.33	48.9 ± 2.78	14.0
Al_50_Yb_50_	0.59 ± 0.35	49.0 ± 2.74	15.0

**Table 5:** Sample of Al-based compositions identified by the genetic algorithm to have simultaneously large predicted *D*_max_ and Δ*T*_x_ values, but, are disregarded due to high price-per-kilogramme

Composition	*D*_max_ (mm)	Δ*T*_x_ (K)	Price ($ per kg)
Al_50_Dy_22_Gd_15_Ce_10_Ir_3_	2.61 ± 0.57	70.2 ± 2.81	4092
Al_50_Nd_40_Rb_7_K_3_	4.18 ± 1.87	30.8 ± 5.07	1186
Al_50_Tm_20_Nd_16_Ho_10_Rb_4_	3.41 ± 0.70	62.8 ± 4.40	1681
Al_50_Tm_29_Tb_11_Dy_8_Y_2_	2.18 ± 1.52	42.6 ± 6.76	1711
Al_60_Yb_31_Gd_5_Rh_4_	4.23 ± 0.76	60.9 ± 4.37	7410
Al_57_Yb_20_Tm_20_Au_3_	2.39 ± 0.55	55.1 ± 4.02	4088

The lanthanide elements have long been incorporated into BMG-forming alloys, including those that are Al-based.^74–80^ Aluminium has a relatively large difference in radius compared to most lanthanides, a key metric under Inoue's rules, which frustrates the growth of crystalline phases during cooling.^66,81,82^ Further, aluminium has large negative mixing enthalpies with the lanthanide elements, promoting mixing rather than phase segregation, another of Inoue's rules.^83^

#### 3.3.2 Copper–zirconium-based alloys

There has long been interest in glass formation in the copper–zirconium alloy system,^84^ and related systems such as Cu–Zr–Al,^85^ Cu–Zr–Ti–(Al),^86^ and Cu–Zr–Ag–Al,^87^ due to their being relatively inexpensive and having favourable mechanical properties in comparison to other BMG-forming alloys.

Here, we probe the copper–zirconium-based alloy space in a number of ways. Firstly, the GA is constrained to search for alloys such that the percentages of Cu and Zr are the two highest in the candidate compositions. Table 6 contains a sample of alloy candidates identified by the GA under these constraints. It is noted that the well-known region of high-GFA around the Zr_50_Cu_50_ composition is identified among the binary alloys. Further, the algorithm identifies beryllium, magnesium, and cerium as important elements to be added to Cu–Zr-based alloys to improve GFA.

**Table 6:** Sample of Cu–Zr-based compositions identified by the genetic algorithm to simultaneously have large predicted *D*_max_ and Δ*T*_x_ values and low price per kilogramme

Composition	*D*_max_ (mm)	Δ*T*_x_ (K)	Price ($ per kg)
Zr_50_Cu_50_	0.89 ± 0.26	52.4 ± 1.71	24.3
Zr_54_Cu_46_	0.80 ± 0.24	51.9 ± 1.81	25.5
Zr_53_Cu_47_	0.80 ± 0.28	52.2 ± 1.55	25.2
Cu_45_Zr_31_Ce_24_	8.26 ± 1.33	80.4 ± 3.67	15.2
Cu_49_Zr_43_Al_8_	7.74 ± 0.83	72.9 ± 2.70	22.7
Zr_50_Cu_39_Be_11_	5.45 ± 2.20	102.5 ± 5.17	38.0
Zr_39_Cu_38_Ce_19_Be_5_	25.98 ± 3.38	99.9 ± 4.99	23.1
Zr_36_Cu_35_Mg_15_Be_14_	18.34 ± 3.70	96.9 ± 8.38	40.4
Zr_49_Cu_31_Be_11_Dy_9_	10.23 ± 1.97	109.0 ± 5.72	88.9
Zr_39_Cu_38_Be_11_Pb_7_Zn_5_	9.80 ± 2.95	92.2 ± 4.91	29.3
Zr_43_Cu_38_La_12_Be_4_Ca_3_	10.40 ± 4.18	89.3 ± 5.44	24.3

**Fig. 7:**
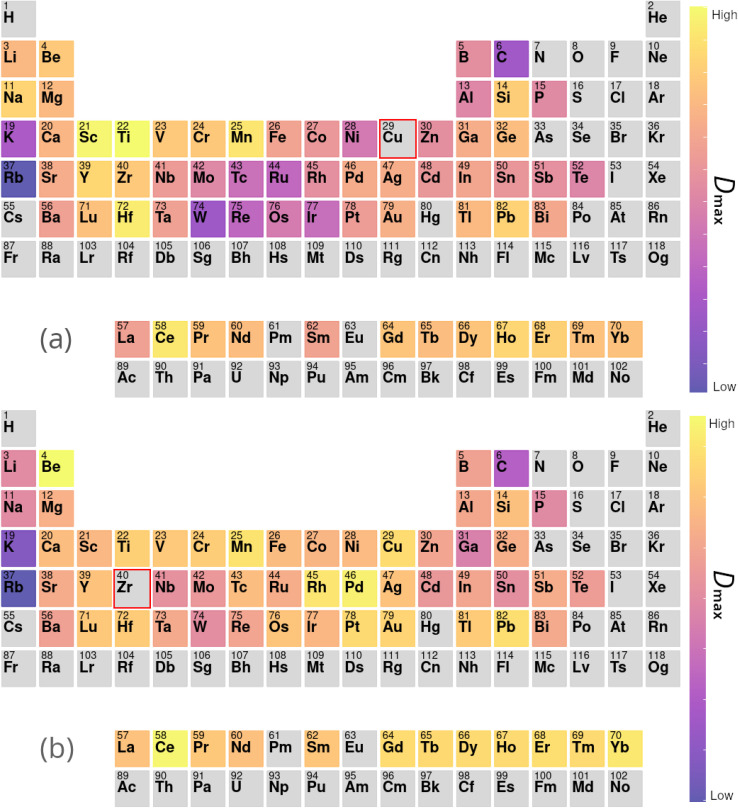
Contributions to *D*_max_ of elements added to (a) copper- and (b) zirconium-based alloys, calculated from the composition-weighted average, defined in eqn (9), for each element across the alloy populations generated by multiple independent runs of the genetic algorithm.

In the second line of investigation, constraints are relaxed such that only one of Cu or Zr is required to be the dominant element in candidate compositions, to allow investigations of alloy systems such as Zr–Ti–Cu–Ni–Be.^88^Fig. 7 demonstrates the average impact on GFA due to the inclusion of each element under these looser constraints, both from the perspective of Cu-dominated and Zr-dominated alloys. Despite dropping the requirement for both Cu and Zr to be present as the dominant components, the GA continues to identify compositions rich in both elements together, implying a high intrinsic GFA associated with the Cu–Zr pairing. However, Fig. 7 also shows a clear difference in the alloying preferences of Cu- and Zr-based BMGs, with each favouring elements from the opposite side of the transition metal series, and Cu exhibiting low GFA when alloyed with certain refractory metals. Both Cu and Zr show high GFA when alloyed with many of the lanthanides, as is also observed with previous evolutions of the GA under different constraints.

### 3.4 Optimization of existing alloys

#### 3.4.1 Zirconium-based glass-forming alloys

**Fig. 8:**
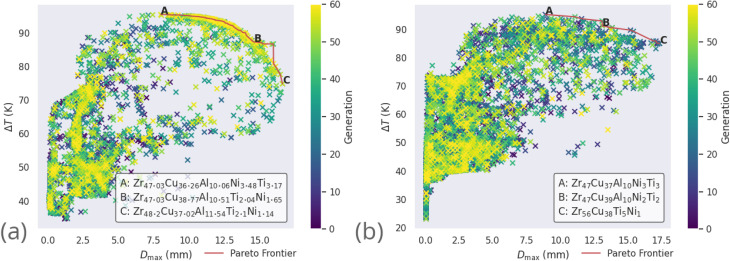
All alloy candidates considered by the genetic algorithm (coloured points) while simultaneously optimizing *D*_max_ and Δ*T*_x_ of the Zr-based alloy compositions (a) LM105 and (b) LM601, and their Pareto frontiers (red).

The particular Zr-based alloys LM105 (Zr_52.5_Ti_5_Cu_17.9_Ni_14.6_Al_10_) and LM601 (Zr_51_Cu_36_Ni_4_Al_9_) were previously identified by Ward *et al.*^5^ for optimization. Fig. 8 shows the *D*_max_–Δ*T*_x_ Pareto frontiers of LM105 and LM601, as predicted by our ensemble neural-network model and explored by the GA. The specific constraints on elemental percentages applied to explore these alloy systems are shown in Table 7, chosen to replicate the investigation parameters of Ward *et al.*

**Table 7:** Constraints on elemental percentages applied to the genetic algorithm to explore the LM105 and LM601 alloy systems

Alloy	Zr (%)	Ti (%)	Cu (%)	Ni (%)	Al (%)	Resolution (%)	Combinations
LM105	47–60	2–6	12–24	0–30	5–15	0.5	181 566
LM601	45–65	0–20	0–30	0–30	0–20	0.5	2 932 786

Alloy candidates found on the Pareto frontier, highlighted in red in Fig. 8, are the optimal set of compositions for the current objectives of maximal *D*_max_ and Δ*T*_x_. For each alloy candidate on the frontier, performance on one of these objectives is degraded by considering another alloy candidate. The alloy used in a real-world application with these objectives would be selected from the Pareto frontier, as all other non-frontier compositions are necessarily inferior.

**Fig. 9:**
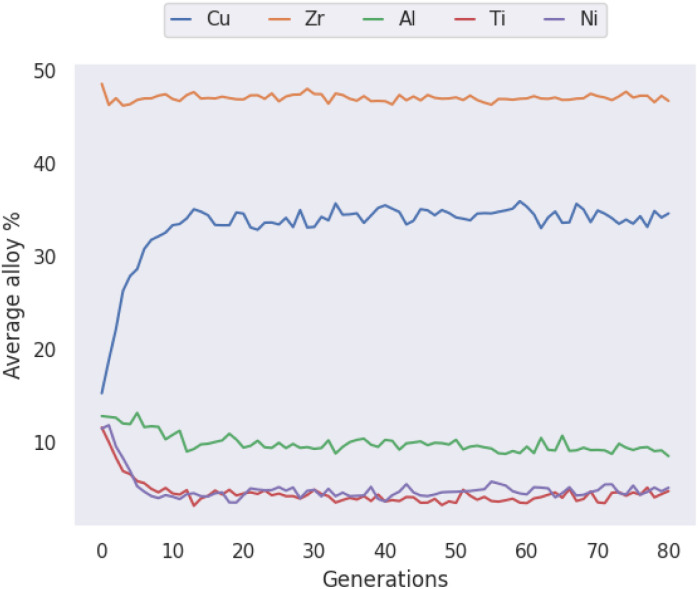
The average atomic percentage of every allowed element across all candidate alloys in each generation of the genetic algorithm when optimizing the LM601 alloy.

The compositions discovered here to be Pareto-optimal are similar to those found by Ward *et al.* Importantly, while the referenced work spent “only a few CPU-days of computer time” performing a brute-force scan of the entire composition space, the present work spent on the order of minutes searching with the GA. The total number of combinations of elemental percentages that sum to 100% in the LM105 case is 181 566, while the presented execution of the genetic algorithm investigates only 12 481 candidates, 6.9% of the composition-space, before converging on solutions. The constraints applied to LM601 are more relaxed, giving a larger space of 2 932 786 possibilities, of which the GA considers 14 684, only 0.5%. This serves to highlight the ability of GAs in general to rapidly identify good solutions to a given problem. In loose analogy, the GA attempts to find and exploit the path of least resistance, in contrast to the brute-force approach. In Fig. 8 multiple ‘clusters’ of solutions are visible, representing different stages of the evolutionary process where the algorithm identifies fruitful regions and explores them for some time, before finding a new region that makes the previous obsolete. Fig. 9 presents similar information on a coarser scale by showing the average percentage of each possible element in all of the alloy candidates at each generation, during evolution of the LM601 alloy. It is shown that the GA quickly increases the amount of Cu, while decreasing the content of the other elements, particularly Al. Following this macroscopic adjustment, the GA enters a period of fine-tuning that is less apparent from the average percentage values, before achieving convergence.

It is also occasionally observed that the GA may identify the solutions deemed to be optimal early in the evolutionary process, and is unable to improve upon them for the remainder of the investigation. This can be seen for optimization of LM106 in Fig. 8, where a large cluster of candidates from later generations (as indicated by the brighter colouring of the points) exists near the frontier, the result of the GA repeatedly attempting to improve upon the Pareto-dominant alloy compositions. While this inability to improve could be due simply to indeed finding the optimal solutions, it may instead indicate that the GA's application of the recombination and mutation operators creates new candidates further away from the parents than would be desired, such that the local minimum is not efficiently explored before the GA moves elsewhere. If this were true, and the ideal solutions are not being found, it is unlikely that repeated independent executions of the GA would return the same results. Here we observe that the GA, when acting on tightly constrained problems, usually returns the same or extremely similar alloy compositions when repeated, indicating that the optimal solutions are indeed being identified. More loosely constrained problems introduce the complexity of there being multiple disparate groups of alloys that perform equally well, with the alloys returned by the algorithm depending largely on the initial population.

While computationally the rate of consideration of candidate solutions may be fast enough to allow the brute-force method in some situations, such as in the example of the Zr-based alloys considered here with fairly strict constraints, in other cases with more elements or relaxed constraints the combinatorial growth of the composition-space renders brute-forcing infeasible.

**Fig. 10:**
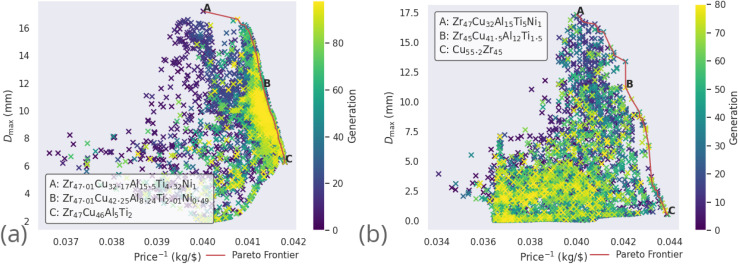
All alloy candidates considered by the genetic algorithm (coloured points) while simultaneously optimizing *D*_max_, Δ*T*_x_, and the price-per-kilogramme of the Zr-based alloy compositions (a) LM105 and (b) LM601, and their Pareto frontiers (red).

Further to the optimization of *D*_max_ and Δ*T*_x_, the alloys LM105 and LM601 are also optimized by the GA to minimize price. Fig. 10 demonstrates the *D*_max_–price plane of the now three-dimensional solution-space. The minimization of price-per-kilogramme is inverted to maximization of kilogrammes-per-dollar for easier conception of the Pareto frontier. The candidate solutions are more densely packed in the region of high kilogrammes-per-dollar and low *D*_max_, due to the GA treating both objectives equally and inexpensive alloys being more common than those with high *D*_max_. In both cases, the GA minimizes the amount of zirconium present in the alloys, as this is the most expensive component. The algorithm also substitutes copper for aluminium when attempting to further reduce the price, but does this at the expense of *D*_max_.

### 3.5 Miscibility of alloy candidates

The energetic favourability of mixing substances is quantified by the mixing enthalpy. In particular if the mixing enthalpy is positive, then the mixing is endothermic, requiring energetic input. In this situation the substances face resistance to mixing, and, at temperatures too low for the entropic term of the Gibbs free energy of mixing to dominate, may not mix at all. While this argument holds for the simple case of binary substance mixing, multi-component mixing introduces the possibility of some components mixing while others do not, with the interaction of each individual pair needing consideration. Here we do not consider this complexity, and use the overall mixing enthalpy as an approximate guide for the miscibility of the alloy as a whole.

Our ensemble neural-network model^18^ was trained on a dataset containing examples of crystalline, GR-forming, and BMG-forming alloys. This resulted in a model proficient in distinguishing between these three categories of alloy, but by nature of the dataset it was not presented with any examples of alloys consisting of immiscible elements. Mixing enthalpy data, which are related to the miscibility, were available during training, but the model could only relate these data to the GFA. However, since a large negative value of the mixing enthalpy is a known criterion for glass-formation under Inoue's rules,^66^ immiscible alloy compositions are likely to be deemed unfavourable by the model.

The Miedema model is applied here to approximate the miscibility of proposed alloy compositions, as a physically grounded check on the output of the GA and machine-learning model. The Miedema model defines the mixing enthalpy in terms of differences in electronegativity and electron density,^89^10
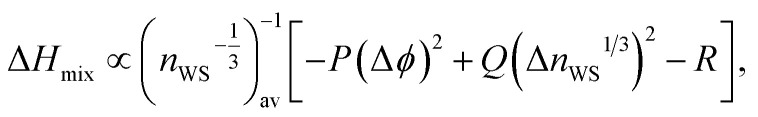
where *n*_WS_ is the electron density at the Wigner–Seitz cell boundary, 
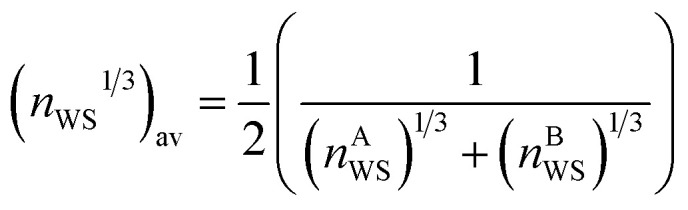
, Δ*n*_WS_^1/3^ = (*n*^A^_WS_)^1/3^ − (*n*^B^_WS_)^1/3^, Δ*Φ* is the difference in electronegativity, and *P*, *Q*, and *R* are empirical constants related to the series of the mixed elements.

**Fig. 11:**
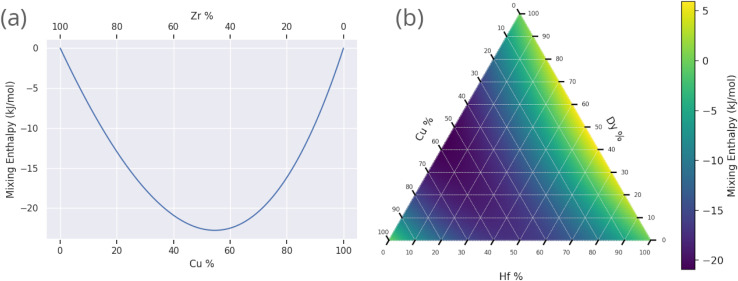
Prediction of the Miedema mixing enthalpy for compositions identified by the genetic algorithm to have large predicted *D*_max_: (a) binary Cu–Zr and (b) ternary Hf–Dy–Cu.

**Fig. 12:**
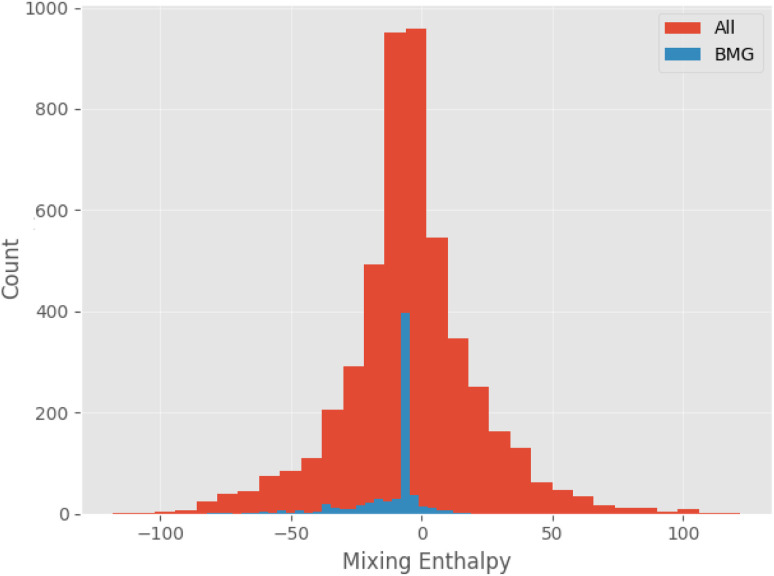
Histogram of mixing enthalpies calculated using the Miedema model applied to candidate compositions generated by the genetic algorithm, comparing the entire population to BMGs alone.

Fig. 11 shows the calculated mixing enthalpy for the novel Mn–Yb and Ti–Rh–Tl compositions, which are identified by the GA to be good glass-formers. The mixing enthalpy of Mn–Yb, while negative across the composition-space, is relatively small, indicating little driving force for mixing. In the case of Ti–Rh–Tl, the majority of the composition-space exhibits a positive enthalpy of mixing, other than in the region of low Tl-content in which the GA identifies candidate alloys with large predicted *D*_max_. Fig. 12 shows the mixing enthalpy throughout the population of alloy candidates identified during an evolution of the GA, demonstrating that the majority of predicted BMG candidates do indeed have a negative mixing enthalpy. However, there remains a small portion of the population with positive mixing enthalpy, which are unlikely to be miscible in reality. This indicates that the alloy compositions identified by the GA, using predictions provided by the neural-network model, may be good glass-formers under the assumption that all combinations of elements can form homogeneous alloys, but other unconsidered factors such as phase-separation could render them difficult to form experimentally.

To incorporate this physical reasoning formally into the alloy selection process, the mixing enthalpy is added as a minimization target for further evolutions of the GA. This has the effect of penalising poor miscibility during evolution, filtering out alloy candidates with poor ability to form homogeneous mixtures. Table 8 contains a sample of alloy candidates identified *via* this process.

**Table 8:** Sample of compositions identified by the genetic algorithm to have large predicted *D*_max_ values and low mixing enthalpies, selected from binary and ternary results

Composition	Predicted *D*_max_ (mm)	Mixing enthalpy (kJ mol^−1^)
Sr_60_Zn_40_	1.70 ± 1.13	−14.21
Cu_46_Zr_54_	0.71 ± 0.29	−22.12
Yb_52_Zn_48_	2.19 ± 0.92	−20.32
Lu_36_Pr_34_Pd_30_	7.86 ± 2.29	−64.62
Y_43_Nd_33_Pd_24_	9.90 ± 2.33	−58.81
Hf_41_Dy_30_Cu_29_	5.27 ± 0.99	−11.95

## 4 Conclusion

Metallic glasses (MGs) have a wealth of potential as a structural and functional materials. Application of MGs to solve real-world problems is, however, limited by the difficulty of discovering novel alloy compositions with high glass-forming ability (GFA). This difficulty originates from the lack of a solid theoretical foundation describing glass formation in metallic systems, and from the intractably large number of possibilities to consider when attempting to discover new glass-forming alloy compositions. Brute-force searches through all possible alloys rapidly become infeasible due to a combinatorial explosion in the number of combinations of elements and atomic percentages. The exhaustive trial-and-error approach that has enabled materials discovery for most of human history cannot be sustained into the future, as materials continue to grow in complexity. In this work, we explore the use of genetic algorithms (GAs) as a solution to the problem in materials discovery of finding the “needle in the haystack”.

GAs search for optimal solutions to any problem through application on a candidate population of the biologically inspired *competition*, *recombination*, and *mutation* operators, requiring direct assessment of only a small fraction of all the possible solutions. It is hoped that the adoption and further development of the GA methodology will lead to reduced costs of materials discovery and development, and to increased usage of BMGs in commercial applications. While this work considers the pairing of a GA with a model for GFA, future investigations may elect to use models for different material properties, such as strength or corrosion resistance.

Here, we pair a GA with a neural-network model for GFA, to enable rapid searching through composition-space for alloys predicted to be excellent glass formers. This is performed both to optimize existing compositions, and to predict entirely novel glass-forming alloys. We target the optimization of the maximum castable diameter of a fully glassy rod, the width of the supercooled region, and the price-per-kilogramme, with the intention of identifying commercially viable novel bulk metallic glass candidates.

Aluminium-based bulk metallic glass (BMG) forming alloys are of interest due to their potential for high strength and corrosion resistance at low cost. The GA identifies many alloy candidates predicted to be good glass-formers, particularly those alloying aluminium with lanthanides. Similar results are obtained for copper–zirconium-based BMG candidates, of interest for their mechanical properties and low relative cost, indicating an important role played by the lanthanide elements in glass formation. Further, application of the GA to optimization of Zr-based alloys returns similar results to previously published brute-force investigations, but with computation time reduced from days to minutes.

## Data availability

The code written during this work implementing the genetic algorithm is available at https://github.com/Robert-Forrest/EvolutionaryDesignOfBMGs.

## Author contributions

Robert M. Forrest: conceptualization, data curation, investigation, methodology, software, writing – original draft, writing – review & editing. A. Lindsay Greer: funding acquisition, supervision, writing – review & editing.

## Conflicts of interest

There are no conflicts to declare.

## Supplementary Material

DD-002-D2DD00078D-s001
