# Supplementary material for: Evolutionary design of machine-learning-predicted bulk metallic glasses
Source: Digit Discov. 2023 Jan 4;2(1):202–18. doi: 10.1039/d2dd00078d (PMC9923804; doi:10.1039/d2dd00078d)
Supplement: DD-002-D2DD00078D-s001 [file DD-002-D2DD00078D-s001.pdf]

Electronic Supplementary Material (ESI) for Digital Discovery.
This journal is © The Royal Society of Chemistry 2023

Supplementary Material

Evolutionary design of machine-learning-predicted bulk metallic glasses

Robert M. Forrest, A. Lindsay Greer

S1 Elemental cost data

Table S1 Cost-per-kilogramme for each element, from multiple sources reporting on different years of market activity.[S1–S4]

Element	Price ($/kg)	Element	Price ($/kg)	Element	Price ($/kg)
H	1.39	Nb	85.6	Tl	4200
He	24	Mo	40.1	Pb	2
Li	85.6	Tc	1e5	Bi	6.36
Be	857	Ru	1.06e4	Po	4.92e13
B	3.68	Rh	1.47e5	At	None
C	0.122	Pd	4.95e4	Rn	None
N	0.14	Ag	521	Fr	None
O	0.154	Cd	2.73	Ra	None
F	2.16	In	167	Ac	2.9e13
Ne	240	Sn	18.7	Th	287
Na	3.43	Sb	5.79	Pa	2.8e5
Mg	2.32	Te	63.5	U	101
Al	1.79	I	35	Np	6.6e5
Si	1.7	Xe	1800	Pu	6.49e6
P	2.69	Cs	6.18e4	Am	7.5e5
S	0.0926	Ba	0.275	Cm	1.6e11
Cl	0.082	La	4.92	Bk	1.85e11
Ar	0.931	Ce	4.71	Cf	1.85e11
K	13.6	Pr	103	Es	None
Ca	2.35	Nd	57.5	Fm	None
Sc	3460	Pm	4.6e5	Md	None
Ti	11.7	Sm	13.9	No	None
V	385	Eu	31.4	Lr	None
Cr	9.4	Gd	28.6	Rf	None
Mn	1.82	Tb	658	Db	None
Fe	0.424	Dy	307	Sg	None
Co	32.8	Ho	57.1	Bh	None
Ni	13.9	Er	26.4	Hs	None
Cu	6	Tm	3000	Mt	None
Zn	2.55	Yb	17.1	Ds	None
Ga	148	Lu	643	Rg	None
Ge	1010	Hf	900	Cn	None
As	1.31	Ta	312	Nh	None
Se	21.4	W	35.3	Fl	None
Br	4.39	Re	4150	Mc	None
Kr	290	Os	1.2e4	Lv	None
Rb	1.55e4	Ir	5.62e4	Ts	None
Sr	6.68	Pt	2.78e4		
Y	31	Hg	30.2		

References

S1 R. K. Zinke and W. H. Werkheiser, *Mineral Commodity Summaries*, United States Geological Survey technical report, 2018.

S2 *CEIC Data*, https://www.ceicdata.com/.

S3 U.S. Energy Information Administration, *Coal Prices and Outlook*, https://www.eia.gov/energyexplained/coal/prices-and-outlook.php.

S4 *Shanghai Metals Market*, metal.com.
